# Advanced Sensing Technologies in Structural Health Monitoring and Its Applications

**DOI:** 10.3390/s25196004

**Published:** 2025-09-29

**Authors:** Ricardo Perera

**Affiliations:** Department of Mechanical Engineering, Universidad Politécnica de Madrid, 28006 Madrid, Spain; ricardo.perera@upm.es; Tel.: +34-910-677-242

## 1. Introduction

Structural Health Monitoring (SHM) is an important area of research due to its strong connection with structural safety and the need to monitor and extend the lifespan of existing structures. In recent years, different technologies and sensing techniques for structural monitoring have rapidly developed. Based on the data extracted from these technologies, SHM algorithms are used to give information and make decisions about structural conditions.

Rapid developments in advanced sensing technologies will help overcome the challenges in realizing smart systems and structures.

This Special Issue focuses on the most recent strategies and developments in innovative sensors and biosensors, as well as their applications in structural monitoring.

## 2. Contributions

The studies published in the current topical collection are briefly summarized below.

The authors of Contribution 1 presented preliminary research on the possibility of using a Honeycomb Sandwich Composite (HSC) model-assisted approach to identify the severity of damage in a composite structure using guided wave propagation. In contrast to full-structure homogenization models, the proposed HSC model was implemented with the actual geometry of the honeycomb core, allowing for visualization.

In Contribution 2, two methods were proposed to address the challenges of small data for SAR (synthetic aperture radar)-based SHM and the long-term variations in civil structures in their inherent properties by environmental and/or operational variability. The first method develops an artificial neural network-based feature normalization by proposing an iterative hyperparameter selection of hidden neurons of the network. The second method is a novel form of unsupervised teacher–student learning that combines an undercomplete deep neural network and an overcomplete single-layer neural network. These methods can effectively deal with the major challenges in the SAR-based SHM applications.

In Contribution 3, a strategy of deep learning-enriched damage detection of metallic pipes with weldments using ultrasonic-guided waves was addressed. Welding defects and material discontinuity damages were detected by the ultrasonic guided waves and identified by eight-layer convolutional neural network (CNN) models with high accuracy. The proposed methodology has yielded highly accurate predictions for damage detection of structures with welding defects in complex situations. The effectiveness and robustness of the proposed method for structure uncertainties using different embedding materials and data under noise interference have also been successfully validated.

In Contribution 4, the applicability and accuracy of a model trained using semi-supervised learning (SSL) rather than supervised learning (SL) with the data from air-coupled impact-echo (IE) tests on concrete structures were analyzed. Features from typical IE data extracted in the frequency domain using principal component analysis (PCA) were used. The accuracy increased by 7–8% compared with SL, and it could categorize good, fair, and poor statuses to higher levels for actual structures.

In Contribution 5, the Optimized Indicators Method was applied to analyze the strain gauge signals, which were recorded during an experimental campaign carried out on a circular instrumented pavement and submitted to accelerated fatigue testing. This method makes it possible to independently evaluate some specific physical characteristics of the pavement using weighting functions. It showed a two-step degradation, starting with damage to the bituminous layer, followed by an alteration in the base layer, which could not be easily deduced from a conventional processing method.

In Contribution 6, a new low-cost triaxial accelerometer based on Arduino technology was presented, Low-cost Adaptable Reliable Accelerometer (LARA), and validated in laboratory experiments. A field test on a footbridge in Barcelona with a span length of 14 m was also carried out using LARA. LARA solved the drawbacks of other accelerometers and showed various advantages compared to previous accelerometers, such as faster sampling frequency, synchronization, and better noise density performance.

Contribution 7 provided a proof-of-concept for using a remotely bonded fiber Bragg grating (FBG) for damage localization. To improve the computational efficiency of the procedure, a particle swarm optimization (PSO)-based algorithm is developed for which an objective function based on an exponential elliptical approach is proposed. The suitability of the PSO for damage localization is checked on a simple aluminum plate, and the performance of the chosen objective function is compared with other objective functions found in the literature.

In Contribution 8, a CNN methodology was proposed to detect the structural damage condition of a building after an earthquake and validated for two currently instrumented essential buildings (Tahara City Hall and Toyohashi Fire Station). The maximum inter-storey drift and absolute acceleration of each storey were used as damage indicators.

The authors of Contribution 9 proposed a novel real-time hybrid simulation (RTHS) control strategy by combining the theories of adaptive and robust control. The control system is designed based on a reformed plant, which is highly simplified compared to the physical plant, without compromising control performance. The proposed strategy is validated by investigating the RTHS benchmark problem of a nonlinear three-story steel frame.

In Contribution 10, the capability of a vibration-related index (E-index) in detecting the degree of the simulated osseointegration process with three lengths of the residual femur (152, 190, and 228 mm) was investigated. A robust assessment method for the osseointegration process is essential to shorten the rehabilitation period and identify the degree of osseointegration prior to the connection of an artificial limb. The findings of this paper highlight that the E-index can be employed as a quantitative justification to assess the degree of the osseointegration process without selecting and tracing the resonant frequency based on the geometry of the residual femur.

In Contribution 11, a multi-bolt loosening identification method based on time-frequency diagrams and a convolutional neural network (CNN) using vibro-acoustic modulation (VAM) signals was proposed. Continuous wavelet transform was employed to obtain the time-frequency diagrams of VAM signals as features. Afterward, the CNN model was trained to intelligently identify the multi-bolt loosening conditions from the raw time-frequency diagrams. The effects of different excitations, CNN models, and dataset sizes were investigated.

In Contribution 12, a wireless and contactless system was applied to evaluate the optimal saw-cutting time based on leaky Rayleigh wave measurements for concrete pavements. The development of sensor networks and the proposed signal processing approach were numerically and experimentally validated with a comparison of saw-cutting procedures. The results demonstrated that the developed wireless system presents identical results to the wired system.

The authors of Contribution 13 previously showed that cold thermography can be a viable defect detection alternative to the most commonly used means of active thermography, known as heating, to detect sub-surface anomalies. Currently, the characterization of defect dimensions, i.e., depth and diameter, has been explored. The results of defect depth prediction were very promising, showing that where active thermography using heating methods cannot be implemented, cold thermography can be a viable alternative.

In Contribution 14, a procedure for the performance assessment of GW-based SHM systems used for monitoring the area around complex structures is explored. The main points of the analysis revolved around the regression models used, the choice of threshold, the damage-path distance dependency, and the influence of the geometrical placement of the path.

Contribution 15 is based on the use of the electromechanical reciprocity theorem to characterize the sensing properties of guided elastic wave transducers. The characterization of the transducer-structure as a transmitter using a Scanning Laser Doppler Vibrometer (SLDV) is straightforward, whereas its characterization as a receiver (sensor) is non-trivial. To solve this issue, electromechanical reciprocity, which is an identity between the transfer functions of electrical-to-mechanical and mechanical-to-electrical conversions, is exploited.

In Contribution 16, the development of a Deep Belief Network (DBN) for tool fault recognition was presented. The network is intended to classify six tool conditions (one healthy and five faulty) in total using image-based vibration signals acquired in real time. The model was designed, trained, tested, and validated through datasets collected considering diverse input parameters.

Contribution 17 provided a new pathway to develop high-performance weigh-in-motion pavement sensors. For this, a road-embedded piezoresistive sensor based on self-sensing nanocomposites has been created. The developed sensors were embedded in a compacted asphalt concrete to validate their applicability to the harsh environment and back-calculate the dynamic vehicle loads on the rutting slab. The results show that the response relationship between the sensor resistance signal and the load is in accordance with the GaussAmp formula.

In Contribution 18, the authors implemented a new measurement protocol in INESSCOM (Integrated Sensor Network for Smart Corrosion Monitoring) to analyze corrosion in reinforced concrete specimens. The protocol allows the identification of the triggering agent of the corrosion process by analyzing the double-layer capacitance of the sensors’ responses. It is extremely useful for simply and quickly determining the precursor corrosion agent, even when the recorded corrosion kinetics are similar.

In Contribution 19, a combined implementation of the Electromechanical Impedance (EMI) technique and Digital Image Correlation (DIC) is implemented to predict early cracks in FRP-strengthened reinforced concrete beams and potentially anticipate the complete failure of the strengthened specimens. An unsupervised hierarchical clustering technique was used to classify the impedance signals. Additionally, strain maps for the different damage stages of the analyzed specimens were evaluated using DIC. The joint application using EMI and DIC more suitably allowed the analysis of the responses of PZT sensors for a better understanding of the failure mechanisms in these types of structures.

In Contribution 20, existing challenges in self-sensing cementitious materials were addressed via the fabrication of self-sensing cementitious sensors using silver nanoparticles (AgNPs), a new type of conductive filler. These sensors exhibited robust pressure-sensitive stability, their stress sensitivity reached a significant value surpassing that of conventional fillers, and their conductive mechanism ensured a stable response to stress. This study provides a significant contribution to addressing the existing challenges in self-sensing cementitious materials and offers a novel reference for further research in this domain.

Contribution 21 presented a ceramic stress sensor with the dimensions of a coin, able to measure the compressive force (stress) applied to its two round faces. The sensor is designed and engineered to be embedded inside concrete or masonry structures, like bridges or buildings. It provides good accuracy, robustness, and simplicity of use at potentially low cost for large-scale applications in civil structures. Moreover, it can be calibrated to the compensated temperature, and it is inherently hermetic, ensuring the protection of sensitive elements from the external environment. It is, therefore, suitable for operating in harsh and dirty environments like civil construction.

The study in Contribution 22 proposed a single UAV for the seismic monitoring and safety assessment of linear infrastructures, along with their computer vision-aided procedures. The proposed procedures were implemented in a full-scale shake-table test of a natural gas pipeline assembly. The investigation into the impact of selecting several parameters for the applied computer vision algorithms on feature matching accuracy is addressed. This study used several validation tests to quantify the effect of the algorithm threshold and dimension selection on matching accuracy.

The work in Contribution 23 presented the design, implementation, and validation of an on-blade sensor system for remote vibration measurement for low-capacity wind turbines. The system was deployed on three wind turbines, with one of them operating in harsh weather conditions, and demonstrated reliable data acquisition and transmission from wind turbines in remote locations, proving the ability to create a fully autonomous system capable of recording data for monitoring and evaluating the state of health of wind turbine blades for extended periods without human intervention.

In Contribution 24, the effect of volume batch during sonication on the electromechanical properties of PVDF-HFP copolymer reinforced with CNTs (carbon nanotubes) or GNPs (graphene nanoplatelets) has been studied. For CNT-based nanocomposites, the DC electrical conductivity decreases with the volume of the batch sonicated. For the GNP-based composites, small batch sizes promoted a pronounced breakage of the nanoparticles due to the excessively aggressive cavitation forces induced by the ultrasound technique, which would explain the lower conductivity values under these conditions.

In Contribution 25, a methodology to identify the structural state without baseline data was proposed. The method is based on the correlation of a small number of sensors using Variational Autoencoder neural networks, and its effectiveness is demonstrated through numerical simulations and experimental structures. The contribution of this study lies in the ability to identify structural damage without baseline data using response data from a small number of sensors, reducing sensor costs and enhancing practical applications in engineering.

The authors of Contribution 26 reported recent advances in bridge SHM backed by smartphone sensor technologies and provided case studies on bridge SHM applications. The review includes model-based and data-driven SHM prospects utilizing smartphones as the sensing and acquisition portal. The review is intended to bring together bridge engineering, SHM, and sensor technology audiences with a decade-long multidisciplinary experience observed within the smartphone-based SHM theme and presents exemplary cases referring to a variety of levels of mobility.

In Contribution 27, a two-sensor technique is used, via both compressive and shear excitations, with a non-iterative rapid data processing method for accurate thickness measurement under an arbitrary time-variant thermal profile. The independent behavior of shear and compressive waves is used to formulate a real-time thickness estimation technique. The developed technique is experimentally validated on a steel plate with fixed acoustic sensors.

Finally, in Contribution 28, the effects of bridge damping on the efficacy of vehicle scanning methods in identifying the modal properties of bridges are investigated. Acceleration responses under different scenarios obtained from a numerical model of a bridge and vehicle are used. The results highlight the importance of correctly simulating the damping behavior of bridges, which is often ignored, to correctly evaluate the efficacy of vehicle scanning methods, and they provide an important stepping stone for future studies in this field.

## 3. Conclusions

In this Special Issue, 28 papers were selected that address different topics related to Advanced Sensing Technologies in Structural Health Monitoring and Its Applications. I hope that the selected articles may provide useful insights into this area of research, inspiring future work and new developments.

I would like to thank all the authors who contributed their work to this Special Issue, as well as the reviewers of the submitted papers who dedicated their time and expertise to providing high-quality reviews that allowed us to finalize a successful Special Issue.

